# β cell aging and age-related diabetes

**DOI:** 10.18632/aging.202593

**Published:** 2021-03-03

**Authors:** Min Zhu, Xiaohong Liu, Wen Liu, Yanrong Lu, Jingqiu Cheng, Younan Chen

**Affiliations:** 1 Key Laboratory of Transplant Engineering and Immunology, NHFPC, Regenerative Medicine Research Center, West China Hospital, Sichuan University, Chengdu, P.R. China; 2 Institutes for Systems Genetics, Frontiers Science Center for Disease-related Molecular Network, West China Hospital, Sichuan University, Chengdu, P.R. China

**Keywords:** aging, type 2 diabetes (T2D), age-related diabetes, β cell function, β cell senescence

## Abstract

Type 2 diabetes is characterized by insulin resistance and loss of β cell mass and function. Aging is considered as a major risk factor for development of type 2 diabetes. However, the roles of pancreatic β cell senescence and systemic aging in the pathogenesis of type 2 diabetes in elderly people remain poorly understood. In this review, we aimed to discuss the current findings and viewpoints focusing on β cell aging and the development of type 2 diabetes.

## INTRODUCTION

Diabetes mellitus is a group of metabolic diseases characterized by hyperglycemia, which results from the defects of insulin secretion, insulin action or both [1–3]. As insulin secreted by islet β cells is the only hypoglycemic hormone in the body, the normal physiological activities of pancreatic islets, particularly β cells, are pivotal for glucose and lipids metabolism in the whole body, and β cell mass loss or function failure would profoundly contribute to diabetes mellitus.

Aging is characterized by gradual functional decline of organs. It is a major risk factor for several chronic diseases, such as cardiovascular disease, stroke, neurodegenerative disorders, as well as diabetes [4, 5]. The 9^th^ edition of International Diabetes Federation (IDF) diabetes Atlas reported that diabetes affects about 136 million people aged 65-99 years in 2019, accounting for 19.3% of the elderly people [6]. China has the largest population of diabetes over the world, and the morbidity of diabetes and prediabetes in people over 60 years old is significantly higher than those under 60 years old, and strikingly, the prevalence of prediabetes in old people is as high as 45.8% [7]. The accumulation of senescent cells in aged organisms is one of the hallmarks of aging [8–10]. Over the past few decades, some studies indicated that the occurrence and progression of type 2 diabetes (T2D) in elderly individuals are tightly associated with senescent β cells, but the specific mechanisms linking systemic aging or cellular senescence with diabetes are still unclear. Here, we review the biology and pathology of pancreatic islets in diabetes, and further discuss the up to date recognition of the relationship between β cell senescence and diabetes.

## The development of pancreas

The pancreas is a highly brunched organ with endocrine and exocrine tissues simultaneously, exerting essential functions for maintenance of body nutrients metabolism homeostasis [11]. The endocrine pancreas is pancreatic islets, mainly composed of five cell types, namely α cells, β cells, δ cells, PP cells and ε cells, responsible for producing glucagon, insulin, somatostatin, pancreatic polypeptide and ghrelin, respectively [12]. There are about 1 million islets in a human pancreas, accounting for 1-2% of the pancreas mass. The majority of islets are located in the body and tail of pancreas, embraced with a dense microvascular network to supply sufficient oxygen and sense the nutrients changes in blood.

The pancreas of human originates as two pancreatic buds, ventral and dorsal bud, from the primitive duodenum. The dorsal bud first appears at the 4^th^ week of gestation, which gives rise to the superior part of the head, neck, body and tail of the pancreas. The ventral bud appears approximately after 30 days of gestation, forming the inferior part of the head of pancreas. With the rotation of stomach and duodenum in the process of embryonic development, the two pancreatic buds fuse tightly at the 7^th^ week of gestation to form the pancreatic ducts [13], which communicate with duodenum through the major duodenal papilla [14]. In contrast to the earlier presence of glucagon producing cells in mouse pancreatic buds, the first endocrine cells appear in human embryo are β cells. The β cells can be detected after around 7-8 weeks of gestation, and represent the main endocrine cell type during the first trimester. Soon after that, the occurrence of α and δ cells are detected at the 8^th^ and 9^th^ week of gestation, respectively [14]. The proportions of α and β cells in pancreas at the 21^st^ week of gestation are similar to that in adult pancreas, while the islets mature after birth [13]. Traditionally, the islets are considered as terminally differentiated and almost impossible to be long-term cultured *in vitro* [15]. However, differentiation of embryonic stem cells (ESCs) or induced pluripotent stem cells (IPSCs) to insulin-secreting cells by mimicking the intercellular signaling during the β cells development, is an ambitious hope for cell therapy in diabetes. More interestingly, recent studies found a group of cells in adult mice and monkeys through single-cell RNA sequencing. Besides able to response to glucose stimulation, these cells express *procr* on cell surface and have progenitor properties, capable to differentiate into all types of islet cells *in vivo*. Furthermore, the authors established an *in vitro* system to generate islet-like organoids, which could be long term expansion and possess similar function and structure to islets *in vivo* [16, 17]. These new findings enlarge our knowledge of islet embryogenesis and give hopes to the regeneration of islets for diabetes in the future.

During the course of embryogenesis, the pancreas development is regulated by multiple signals and transcription factors at different stages, in which signals from notochord, mesenchyme, epithelia and endothelia are essential for the initiation of pancreas development and exocrine or endocrine cell fate decision. Importantly, some transcription factors play a dominant role in the generation of fetal islet. The transcription factor pancreatic and duodenal homeobox 1 (PDX-1 also known as IPF-1, IDX-1, STF-1 or IUF-1) is a marker of pancreatic progenitor cells at early pancreas development stage, playing a crucial role in the growth, differentiation and morphogenesis of pancreas development [18]. PDX-1 can be detected at the fourth week of human gestation [11]. Researches have shown that mice lack of PDX-1 fail to form pancreas and die a few days after birth [13, 19]. Neurogenin (also called Neurog3 or Ngn3) is a basic-helix-loop-helix (bHLH) protein, which is transiently required for the endocrine cell fate decision during pancreas development. The expression level of Ngn3 reaches the peak around 12 weeks post-conception, but it is not detected in human fetal after 35 weeks of gestation [12]. Researches indicate that loss or mutation of Ngn3 in mice cause absence of endocrine cells in pancreas and intestinal, resulting in diabetes, congenital diarrhea, or nutrients absorption failure [20]. In addition, Ngn3 plays a key role in β cell differentiation through regulating multiple target genes required for pancreatic fate. NeuroD1, Pax6, Arx, Nkx2.2 and MNX1 are the downstream transcription factors of Ngn3, being identified as causative factors for permanent neonatal diabetes mellitus (PNDM) when they are mutated. Other transcription factors critical for pancreas development include PTF1A, Sox9, GATA4/GATA6, Nkx6.1, Hes1 and others, and the details of these factors are not included in this review.

In the last two decades, more and more attentions have been drawn on the causative link between suboptimal pancreas development and T2D risk. Interesting results reported by Hardikar et al. revealed that undernourished rats with low birth weight for over 50 generations display epigenetic modifications, leading to significant decrease of PDX1 binding at insulin-2 gene promoter. And this change is associated with increased metabolic abnormality and diabetes in adults, and is unable to be reversed following nutrient recuperation for two generations [21]. De Rooij et al. demonstrated that non-diabetic people prenatally exposed to famine during the *Dutch Hunger Winter* of 1944–1945 present impaired insulin secretion and glucose tolerance, and people exposed to famine at midgestation have a significant lower disposition index, compared with people unexposed to famine. But these phenomena did not appear in people experiencing famine after 32 weeks of gestation [22]. The time points greatly match to the profile of Ngn3 expression during embryonic pancreas development. These striking findings suggest that epigenetic changes in fetal pancreas programming might get transgenerational effects on adult metabolic risks, which might be an explanation for the explosive increase of diabetes in developing countries in the last decade.

## β cell dysfunction in type 2 diabetes

**Figure 1:**
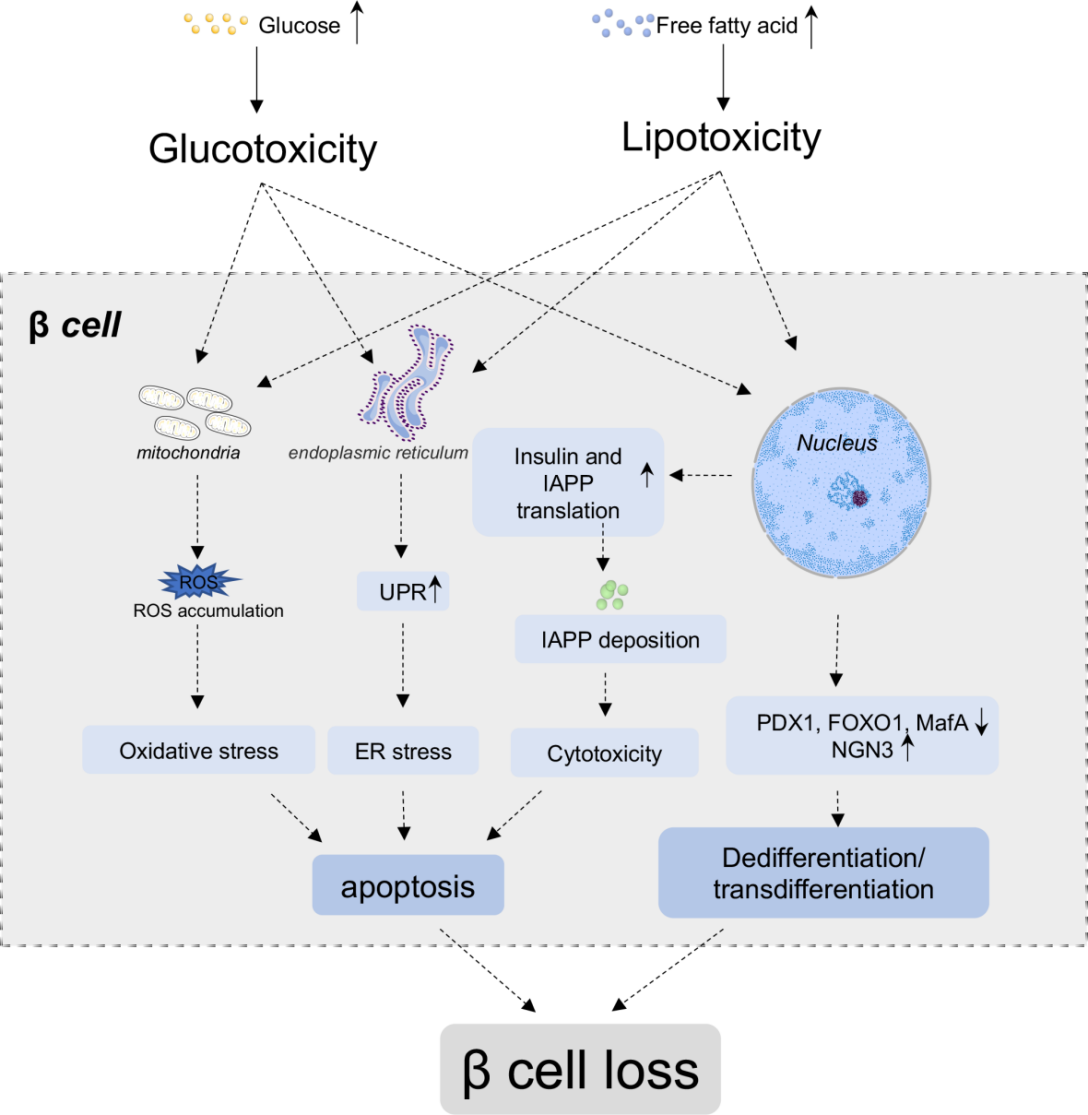
**β cell loss in T2D.** In normal conditions, glucose and nutrients influx stimulate β cells to secrete insulin to cope with increased blood glucose level. Under chronic high blood glucose and high free fatty acids condition, β cells secrete more insulin to compensate hyperglycemia, while simultaneously, co-secrete more islets amyloid polypeptide (IAPP). The deposition of IAPP further aggregate into amyloid plaque in β cells. Glucotoxicity, lipotoxicity and amyloid deposit lead to the accumulation of reactive oxygen species (ROS), unfolded protein and so on, which results in oxidative stress, ER stress, inflammation and other cytotoxicity of β cells, and ultimately induces apoptosis of β cells. Additionally, high glucose and lipid lead to the downregulation of critical transcriptional factors, such as PDX1, FoxO1 and MafA, and re-express of progenitor marker Ngn3, which lead to the dedifferentiation and/or transdifferentiation of β cells. Both the apoptosis and dedifferentiation/transdifferentiation could contribute the mass loss of β cells.

Insulin is a low molecular weight protein secreted by β cells of pancreatic islets. Insulin plays a central role in glucose homeostasis. The secretion of insulin is regulated by blood nutrients, hormones, as well as nerves, among which, glucose is the prime modulator. Glucose enters the β cells through GLUT1 (GLUT2 in rodent) on the cell membrane, then, participates in citric acid cycle and causes a rapid increased ratio of ATP to ADP, which further reduces the K^+^ efflux through inhibiting an ATP-sensitive K^+^ channel, leading to the depolarization of cell membrane and activation of a voltage-gated Ca^2+^ channel to promote Ca^2+^ influx. The increased intracellular Ca^2+^ triggers insulin granule exocytosis from β cells into blood circulation [23]. Once insufficient insulin secretion fails to compensate the high demand for insulin, the hyperglycemia occurs. β cell mass loss is a prominent feature in diabetes (Figure 1). Piles of evidence demonstrates that long term exposure to hyperglycemia and hyperlipidemia results in glucotoxicity and lipotoxicity in β cells, which lead to the gradually deterioration of their function and loss of β cell mass, and ultimately aggravation of diabetic state [24, 25]. The β cell defects in T2D are comprehensively studied in the last two decades. The traditional recognition of its underlying mechanisms suggests that endoplasmic reticulum (ER) and oxidative stress, chronic inflammation and mitochondrial dysfunction are the major aspects responsible for the impairments of β cell expansion, insulin production and secretion, and final β cell apoptosis in T2D. But recently, increasing evidence indicates that the inherent β cell plasticity may play an important role in the development of T2D, including dedifferentiation and transdifferentiation [26].

## β cell apoptosis

Studies of pancreatic samples from T2D patients and rodent models reported that β cell mass decreases up to 60%, and they found β cell apoptosis is predominantly responsive for the β cells loss in the progression of T2D [27, 28].

Apoptosis is a form of programmed cell death, which is implicated in the normal physiology of pancreatic development and final β cell mass formation [29]. The dynamic β cell mass is determined by the balance between β cell replication or neogenesis and apoptosis [30]. Glucotoxicity, lipotoxicity and islet amyloid polypeptide (IAPP) are the primary causative factors for increased β cell apoptosis in T2D, while ER stress and oxidative stress, as well as autophagy could be the link between upstream metabolic stimuli and downstream apoptotic machinery [31].

### Glucotoxicity and β cell apoptosis

Oxidative stress is extensively involved in many pathologies, including the development of β cell dysfunction and insulin resistance, and it is the most commonly accepted mechanism of β cell dysfunction caused by hyperglycemia [32]. Hyperglycemia activates intrinsic apoptosis pathway of β cells by increasing oxidative and nitrosative stress, as well as crosstalk between proapoptotic Bcl-2 family members and caspase cascade. β cells are particularly sensitive to ROS, because they contain low content of intrinsic antioxidant proteins [33]. It is shown that low grade of ROS is beneficial to stimulate insulin secrete [34], on the contrary, prolonged glucotoxicity results in free-radical damage to DNA, protein, and lipids, and increases apoptosis in β cells as a consequence [35].

High glucose increases ROS production by 1) activating NADPH oxidase on the cell membrane; 2) interrupting mitochondrial electron transport chain at complex III; and 3) impairing antioxidant system including SOD, GPx and CAT. ROS accumulation in mitochondria is a main cause of mitochondrial dysfunction [36]. It results in the formation of lipid peroxides, imbalance of mitochondrial fission and fusion, interruption of mito-autophagy process and importantly block of transduction of signals coupling glucose metabolism to insulin secretion. Oxidative stress in β cells also increases a series of proinflammatory cytokines (IFN-γ, IL-1β, TNF-α, etc). These changes in cytokine profile contribute to the activation of the key transcription factors, nuclear factor (NF)-kB and STAT-1, and consequently the expression of Fas and TNFaR1, responsible for the extrinsic pathway of apoptosis [37].

Recently, it has been found that the decreased expressions of transcription factors PDX1, MafA, FoxO1, and Nkx6.1 in T2D might be associated with β cell apoptosis. As PDX1 is important for islets development and adult β cell survival, its complete deficiency results in pancreatic agenesis, leading to increased β cell dysfunction and death [38]. Research indicated that its partial deficiency increases islets apoptosis in a PDX1^+/-^ mice model [39]. Antioxidants treatment in high fat/high sucrose fed mice model and INS-1 cells exposed to high glucose and lipid could enhance the PDX1 level and improve apoptosis of β cells [25].

### Lipotoxicity and β cell apoptosis

Besides high level of blood glucose, hyperlipidemia is also a major characteristic of T2D. It is well known that saturated fatty acids show strong toxicity to pancreatic β cells. Our data demonstrated that exposure to palmitic acid for 24 hours results in substantial apoptosis and elevated ER stress in INS-1 cells. However, unsaturated fatty acids do not show toxicity and further rescue cell viability when treated with palmitate together [40]. A great amount of evidence suggests that ER stress is the most important link between lipotoxicity and β cell dysfunction or cell death [41, 42]. ER stress activates inositol-requiring protein 1 α (IRE1α), which activates c-Jun N-terminal kinase (JNK) via phosphorylation [43]. JNK can inhibit the anti-apoptotic properties via suppressing the expression of Bcl-2 family while upregulating the pro-apoptotic protein BAX to induce mitochondria-mediated apoptosis [44]. Apart from JNK pathway, the activations of protein kinase C (PKC), p38 mitogen-activated protein kinase (p38 MAPK), extracellular signal-regulated kinase 1/2 (ERK1/2), NLRP3 inflammasome, and Akt (also known as protein kinase B (PKB) kinase) signals have also contributed to fatty acids induced lipoapoptosis [43].

It is worthy to mention that ceramide, the metabolite of palmitate, is also a key component of apoptosis signal transduction. It is considered as a critical contributor to lipoapoptosis. Ceramide promotes lipid raft fusion, which promotes the clustering of death receptors, and allows the recruitment of various apoptotic proteins to the receptors. As a consequence, the activations of adapter proteins such as Fas-associated death domain (FADD) and tumor necrosis factor receptor type 1-associated death domain (TRADD) trigger the caspase cascade. In addition, ceramide can activate NFκB to increase inducible nitric oxide synthase, and result in an increase in nitric oxide generation, and ultimately increase of apoptosis [45].

### IAPP and β cell apoptosis

Islet amyloid polypeptide (IAPP, or Amylin), is a 37-aa residues long peptide neuroendocrine hormone, which is co-secreted with insulin from pancreatic β cells. Due to the presence of a hydrophobic amino acid proline in the middle region of IAPP, human IAPP is prone to aggregate into insoluble amyloid plaque. Previous studies indicated that the polypeptide involved in the regulate of glucose homeostasis, but the function of IAPP remains not fully understood [46, 47]. It was shown that IAPP inhibits the secretion of insulin and suppress the insulin-stimulated glucose transport *in vitro* by a post-insulin-receptor effect. [46, 48]. Studies of human pancreas from people with or without T2D showed that β cell loss and apoptosis is associated with the islet amyloid deposition [49, 50]. Previous studies demonstrated that the deposition of amyloid induces ER stress, and the ER stress inhibitor 4-phenylbutyrate (PBA) can alleviate β cell apoptosis [51]. Westermark P. demonstrated that β cells nearby amyloid are penetrated by bundles of fibrils [52]. In addition, Kawahara M. and colleagues have shown that the amyloid affects β cell function through interaction with β cell membrane, leading to upregulated Ca^2+^ influx, increased oxidative and ER stress [53], and further activated apoptosis via JNK and caspase pathways [54]. Taken together, how IAPP damages β cells is still elusive, but it is likely to involve in multiple pathological processes and affect β cell functionality in different ways.

## Loss of β cell identity

The loss of β cell has commonly been proposed to be attributable to cell apoptosis. But recently, this concept has been challenged by some new ideas, that the deficit of β cell mass may be due to aberrant dedifferentiation and transdifferentiation of β cells [55, 56]. Adult mature β cells express a series of transcription factors to maintain their identity, including PDX1, MafA, Nkx6.1, Nkx2.2 and FoxO1. Dedifferentiation is a state that cell changes its gene expression profile accompanied by the loss of its phenotype and back to a more immature state. Meanwhile, transdifferentiation is defined as the transformation of one terminally differentiated cell type into another without reverting to a more primitive progenitor-like state [27].

MafA is the last identity transcription factor appearing in mature β cells, inactivation of which impairs β cell function and glucose stimulated insulin secretion (GSIS) without mass loss [57]. It has been found that the loss of MafA in β cells is one of the earliest feature in T2D of human and rodents [58]. In adult mouse β cells, gradual loss of β cell identity transcription factors, such as PDX1 and FoxO1, leads to β cell re-expresses the endocrine progenitor markers [58, 59]. Likewise, the loss of PDX1 leads to express glucagon and α cell identity transcription factor Arx in the mice β cells. A study of human pancreas has found some evidence of dedifferentiation and transdifferentiation in islets. They reported that in T2D donors, there were increased expression of β cell markers in glucagon or somatostatin positive cells, which suggests the loss of lineage specific identity in some islet cells [60]. These alterations cause β cell reconfiguration in structure and function ultimately.

FoxO1 is a multifunctional transcription factor in β cells, which inhibits β cell proliferation and promotes apoptosis, while resists oxidative stress via the induction of antioxidant enzymes. FoxO1 also promotes the differentiation of pancreatic cells [61]. In normal conditions, FoxO1 is localized in cytoplasm of β cells in response to insulin stimulation. FoxO1 binds to PDX1 promoter competitively with FoxA2, a crucial transcription factor for pancreas development, to inhibit β cell proliferation. In hyperglycemia conditions, FoxO1 could be found in nucleus or even losing its expression, to response oxidative stress, which is associated with the loss of insulin secretion [62]. Kobayashi et al. found that β-cell-specific FoxO1 knockout mice exhibit loss of β cell mass and correspondingly elevation of α cell number under increased physiological demand conditions. Moreover, these FoxO1-deficient β cells do not express PDX1 and MafA, but re-express progenitor marker Ngn3 [63]. Nilsson et al. found that the FoxO1-deficient β cells express glucagon [64], suggesting the dedifferentiation occurs in these mice.

β cell is a specialized cell type distinct from other cells in terms of function and physiological responsibility. Its identity maintenance is not only the consequence of expressions of β cell unique genes, but also the selective repression of some housekeeping genes [65]. There have been identified at least 60 disallowed genes of β cells, such as monocarboxylate carrier 1 (MCT1), lactate dehydrogenase A (LDHA), low Km hexokinase I (HKI) and repressor element 1 silencing transcription factor (REST). Overexpression of these disallowed genes results in destruct of GSIS and proper insulin secretion, and β cell mass loss and dysfunction may be the fate of these cells [66, 67].

## Aging and diabetes

Aging is a result of accumulation of cellular damage, which leads to the function decline of tissues and organs over time [8]. The previous studies suggested the β cell dysfunction with age is mainly caused by the loss of functional β cell mass. However, in recent years, some researchers have put forward different opinions, suggesting that the intrinsic function of β cells does not significantly change with age, but their fully functioning is affected by the systemic senescent environment [68, 69].

## Age-related decrease in functional β-cell mass

### Replication and proliferation

With age, the replicative ability of most organs and tissues decreases, including pancreatic islets. β cell replication is the primary mechanism of postnatal β cell mass expansion in human [30]. Age-related decline of the proliferation and replication potentials in β cells has been well studied, and it is proposed as a cause of increased prevalence of T2D in the elderly. Numerous studies found that the expressions of cell cycle activators decrease, while the expressions of cell cycle inhibitors increase in senescent β cells [70, 71]. p16^INK4a^ is a tumor suppressor protein and cyclin kinase inhibitor, playing its role through preventing the phosphorylation of the retinoblastoma (Rb) protein by inhibition of the combination of Cyclin D and CDK4/CDK6 [72]. The expression of p16^INK4a^ is a biomarker of aging [73], and it is upregulated in β cells with advanced age, which contributes to cell cycle arrest and cellular senescence in mice and human [74]. Furthermore, p16^INK4a^ also participates in β cell regeneration. It is well known that β cells exhibit some extent of regeneration capability when undergoing sever damage [75]. In streptozotocin (STZ) induced diabetic mice models, STZ injection led to β cell destruction in all the animals, in which p16^INK4a-/-^ mice showed much lower blood glucose levels in comparison with the persistent hyperglycemia in p16^INK4a+/+^ and p16^INK4a+/-^ groups, suggesting that the β cells in p16^INK4a-/-^ mice probably have better regeneration capacity after STZ damage. Then they hypothesized that p16^INK4a^ is involved in the inhibition of β cell regeneration [70]. Another study in Wistar rats experiencing partial pancreatectomy (Px) found that young rats after Px exhibited an increased β cell mass, on the contrary, middle-age animals did not change in β cell mass, implying the decline of regenerative capacity with age [76]. Moreover, PDX1 is also an important marker of β cell replication. Studies have shown that the expression of PDX1 in β cells decreases with advancing age in rat and human, and the replication ability of human β cells is tightly coupled with PDX1 expression [77]. Moreover, pancreas tissue of aged Wistar rats showed an age-related decrease of replication ability, evidenced by the less positive staining of proliferation marker Ki67 and proliferating cell nuclear antigen (PCNA) [78]. Platelet-derived growth factor receptor (PDGFR) signaling is essential for proliferation, migration and survival of several cell types including β cells. The activation of PDGFR stimulates DNA synthesis of islets *in vitro* [79], and regulates β cell proliferation through a Ezh2 dependent way, which can repress p16 expression in β cells. The deficiency of PDGFR signal coupled with decreased Ezh2 and increased p16^INK4a^ has been seen in a RIP-Cre; Pdgfra^f/f^ mice. Consistently, PDGFR signal expression is decreased in pancreatic islets of old mice as well as adult humans [80]. These data indicate that the proliferation and regeneration of β cells are decreased with aging, which might be attributable to the upregulation of cell cycle inhibitors and diminish of proliferative signals. Nevertheless, the studies on whether the replication and apoptosis of β cells decreasing with advancing age are limited, and its underlying mechanisms are still unclear and controversial.

### Disturbance of transcriptional and protein homeostasis

In the last couple of decades, a variety of molecular pathological alterations have been identified in aging process, including DNA damage, altered DNA methylation patterns, and compromised transcriptional stability, resulting in deteriorated genomic stability, epigenetic disturbance and transcriptional noise throughout life [10]. Previous study has demonstrated that the accumulation of DNA mutations is associated with decreased transcriptional stability [81], and the mutation signature is closely related to oxidative stress and ROS dependent lesion on DNA. Theoretically, these genetic aberrances may participate in the process of age-related diabetes as well. Recently, a single cell analysis of eight human pancreas from different ages demonstrated that islets from older donors display increased transcriptional noise, genetic errors and potential fate drift [82]. It is found that islets from old donors represent a specific mutation signature, including a higher CDKN2A gene expression and abnormal high fraction of atypical α or β cells co-expressing insulin and glucagon. In addition, they found 8-hydroxyguanosine levels are markedly elevated in β cell DNA compared to non-islet cells, indicating oxidation of guanosine might drive the mutational signature of β cells, which probably interprets the link between oxidative damage and endocrine dysfunction in the pathology of T2D [82].

In addition to transcriptional stability, loss of proteostasis has also been found in aging. Protein homeostasis is pivotal for normal cell function. Therefore, the gradual compromised protein degradation and clearance in the process of aging lead to a disturbance of cellular proteostasis, accompanied with oxidative stress, ER stress and even cell death, which is proved to be a hallmark of aging and age-related diseases. More recently, a single cell sequencing of young and old non-human primate pancreatic islets portrays a transcriptomic landscape of aged α and β cells. This study is consistent with the above human pancreases sequencing, showing that endocrine cells age heterogeneously, and the transcriptional noise increases with aging. Interestingly, the transcriptional noise is only increased in aged α and β cells, rather than other islet cell types, implying α and β cells are more vulnerable to aging. Further analysis of differentially expressed genes showed that the unfolded protein response (UPR) is a main specifically altered pathway in aged β cells, indicating the occurrence of disrupted proteostasis. And aging-associated genes analysis found that escalated UPR genes in β cells mainly concentrate upon ATF6 and IRE1 branches, especially an ER chaperone, HSP90B1 [17].

Oxidative stress is a hallmark of aging as well as diabetes, and the accumulation of ROS leads to the formation of advanced glycation end products (AGEs) and disturbed proteostasis [83]. AGEs are an intermediate of glycolysis and lipid peroxidation. It is suggested that hyperglycemia accelerates the formation and deposition of AGEs, nevertheless, it also occurs in aging [68]. After binding to the receptor of advanced glycation end products (RAGE), AGEs activate NADPH oxidases, leading to the augment of ROS production, and proinflammatory response in β cells [84], and the increased ROS in turn results in the formation of AGEs [85]. In addition to AGEs, IAPP accumulation also increases with age, contributing to the disturbance of cellular proteostasis [86]. In the study of human pancreas from autopsies of Japanese showed that the deposition of IAPP increase with age, and the ratio of IAPP deposition for patients without diabetes but older than 70 years showed a significant increase (41.23%) compared with that for patients younger than 70 years (5.98%) [87].

### Cellular senescence

Cellular senescence is a state of cell cycle arrest of previously replication-competent cells but remain metabolically active [9, 88, 89]. Senescent cells always display increased cell size and lysosomal β-galactosidase activity, characterized by activation of chronic DNA damage response (DDR), enhanced expressions of cyclin-dependent kinase inhibitors and apoptosis resistance. In addition, senescent cells secret senescence-associated secretory phenotype (SASP), including a group of proinflammatory cytokines, chemokines and extracellular matrix factors et al. [88]. Cellular senescence is a fundamental mechanism of aging and accumulation of senescent cells is considered as a main feature of aged organisms [8, 9, 88]. It is reported that β cell senescence may not only be predominantly involved in the mechanisms of age-related diabetes, but also contribute to diabetes progress in younger population. There is strong evidence showing that the senescent β cell burden is increased in both type 1 diabetes (T1D) and T2D. A cross-sectional study of 80 human pancreatic tissues showed that compared with non-diabetes (ND) and impaired fasting glucose (IFG) individuals, islets from T2D donors have a significant higher proportion of senescent β cells [90]. Recent study indicated that a great proportion of β cells in NOD mice and human T1D become senescent. These cells typically secrete SASP, not only stimulating monocytes chemotaxis, but also inducing normal β cells senescent through paracrine effect of primary senescent β cells. Moreover, senescent β cells upregulate Bcl-2, and then induce apoptosis resistance, resulting in the accumulation of senescent β cells in islets, which in turn detrimentally affects healthy β cells through SASP effect [91]. Aguayo et al. found that both old individuals and T2D patients have a higher proportion of senescent β cells, which was also confirmed in rodent models. Further analysis through single cell sequencing of aged mice found that senescent β cells exhibit a downregulation of its identity genes, including PDX1 and Nkx6.1 et al., and an upregulation of disallowed genes and aging markers, such as ldha and p16^INK4a^. Both in high fat diet (HFD) and transgenic INK-ATTAC mice model, insulin resistance induces the appearance of senescent β cells, and aggravates the state of insulin resistance, leading to the onset of diabetes ultimately [92]. The authors indicated that senolytic drugs could clear these senescent cells, improve β cell function and prevent disease progression in T1D or T2D. Moreover, in the recent study that conducted a single-cell sequencing of non-diabetic young and aged cynomolgus monkeys, the results were in agreement with the above studies of mice and human. The study demonstrated that there are increased senescence markers CDKN1A, CDKN2A and SASP expressed in aged α and β cells, implying that pancreatic islets of aged monkeys are enriched with senescent cells [17]. Another study of Aguayo showed that the β cells are dysfunctional during aging in mice. They found an aging marker of β cells, IGF1R, whose expression is increased in aging and T2D rodents and human, and additionally, its expression is accelerated by p16^INK4a^ expression and insulin resistance state [93]. Studies have shown that p16^INK4a^ overexpressed β cells present enhanced insulin secretion while limited proliferative potential, which indicates that the senescent β cells are in a compensatory state [74]. Taken together, these studies indicate that senescence of β cells act as a common contributor in the etiology of T1D and T2D, and the accumulating of senescent β cells in islets with aging may contribute to the failure of β cell function.

### Altered β cell function, insulin secretion and insulin sensitivity

The progressive decline of glucose tolerance with advanced age has been identified as the important pathogenesis of T2D, attributable to peripheral insulin resistance as well as the defect of β cell function. Some studies have shown that in old people, the insulin secretion is compromised, accompanied with decreased insulin clearance rate [94] and increased circulating proinsulin concentration [95], which may be the potential explanation of age-related hyperglycemia. However, the mechanisms of aging driven glucose intolerance and insulin resistance are still unclear. A study showed that there are similar first phase insulin secretion and insulin sensitivity in old and young people, but old people exhibit a decreased second phase insulin secretion [96]. Previous *in vivo* and *in vitro* studies demonstrated that in human and rodent without T2D, old islets secret more insulin in respond to the glucose challenge, suggesting a decline of insulin sensitivity in the course of aging. The glucose elevation stimulates release of insulin granule via sensing the increased ATP/ADP ratio, which inhibits the K^+^ efflux and further promotes Ca^2+^ influx. A great bunch of studies revealed that proper mitochondrial function is essential for intracellular Ca^2+^ signaling as well as insulin secretion. The mtDNA mutation mice model, in which the mitochondria are disrupted, present impaired Ca^2+^ signaling of β cells with aging, leading to a compromised insulin secretion capability both *in vivo* and *in vitro*. Furthermore, an analysis of human islets indicated that the coordination of Ca^2+^ dynamics, gap junction and insulin secretion dynamic of β cells are weakening with age [97]. Another study showed that in old mice β cells are dysfunctional, exhibiting high basal insulin secretion with high fasting blood glucose, but insufficient insulin secretion under high glucose stimulation. The author sorted β cells into three subpopulations: GFP^low^, GFP^medium^, and GFP^high^ in a transgenic C57BL/6J MIP:GFP mice model, in which GFP expression is driven by the insulin promoter. They found the number of GFP^low^ β cells are higher in young mice, but in contrast, the GFP^high^ β cells are more in old mice, indicating the β cell heterogeneity and insulin secretion are changed in old mice, which also supports the finding that β cells in aged mice have a high basal insulin secretion but impaired glucose response ability [93]. Despite of islets dysfunction, the decline of peripheral insulin sensitivity with aging has been observed in rodents and human [98, 99], and more recently, hyperinsulinemic-euglycemic clamp of mice demonstrated that aged mice on both chow and high-fat diets present an impaired insulin sensitivity and declined insulin clearance, which is consistent with human results, indicating that it is related to age but independent with adiposity [100].

## Abnormality of non-β cells

Many researchers have held the viewpoint that β cell function decreasing with advancing age, is the main cause of the high morbidity of age-related diabetes. However, some studies draw different conclusions, suggesting that the intrinsic decline of β cell function with age is so limited, and may not the predominant cause of age-related diabetes, but the non-β cells in islets and even in other aged organs or tissues, as well as aged microenvironment may play an important role in the pathogenesis of age-related diabetes. A study of aged mice showed that there are increased AGEs and p16^INK4a^ expressing in pancreas with aging, but the deposition of AGEs is obvious in blood vessels rather than endocrine cells in islets. Consistently, ROS production and expressions of iNOS and 3-NT are upregulated in aged mice, while they are also located in the blood vessels of pancreatic islets [68]. In another research, to elucidate whether β cell function declines with age, Joana et al. isolated islets from non-diabetic, young or old mice and human, and found that islets from old mice and human remain glucose-sensitive and capable to secrete enough insulin to response glucose stimulation, but the glucose tolerance is impaired in old mice. More importantly, the blood vessels of islets in old mice and human are inflamed and fibrotic, suggesting that either glucose homeostasis or microenvironment homeostasis in islets are disturbed in old mice. Furthermore, the authors transplanted islets from non-diabetic mature young or old mice into the anterior chamber of STZ-induced diabetic young or old mice. After transplantation, blood glucose returned to normal in both young recipient mice with young or aged islets, but old hosts with aged islets reversed diabetes only in half of the recipients under a prolonged time. Monitoring the old grafts in young hosts found that the grafts were well revascularized after transplantation [69]. This experiment perfectly demonstrated that with advanced age, the β cell function may has little intrinsic decline, which is insufficient to drive the onset of T2D, however, islet vascular aging is supposed to play a key role in the age-related β cell dysfunction. These researches suggest that in the process of aging, the β cells may still functional, but their function is affected by the system senescent milieu, which provides a new insight to age-related diabetes mechanism.

## CONCLUSIONS

**Figure 2:**
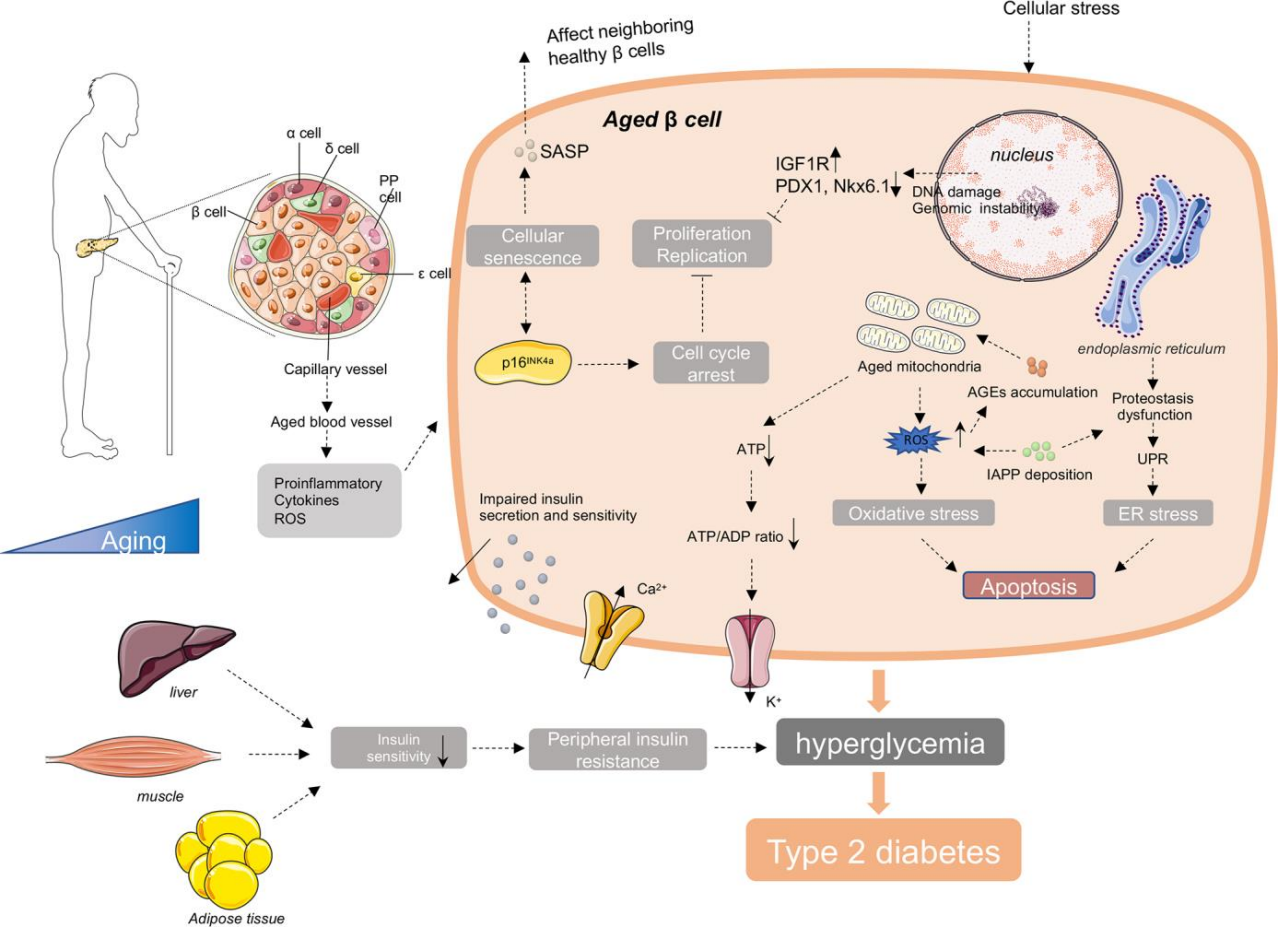
**Molecular mechanisms involving in age-related diabetes.** In the process of aging, there are increased accumulation of ROS, unfolded protein, DNA damage, IAPP, AGEs and other cellular stress in aged β cells. These intracellular stresses make β cells more susceptible to apoptosis. And with age, there are increased cell cycle inhibitors, such as p16INK4a, and decreased cell cycle activators, such as CDK4 and CDK6 in β cells. These changes lead to the reduce of the proliferation and regeneration potential and induce the cellular senescence of β cells. Senescent β cells secrete a series of senescence-associated secretory phenotype (SASP), promoting the senescence of neighboring healthy β cells through induction of paracrine senescence. In addition, islet blood vessels are undergoing aging as well. Oxidative damage, inflammation and fibrosis in islet blood vessels may disturb β cell function. Besides islets, the external factors include the reduction of insulin sensitivity in peripheral insulin responsive tissues with advanced age, responsible for the increased demand for insulin and final exhaustion of β cells. Taken together, the decreased insulin secretion capacity of β cells and increased insulin resistance with age lead to the failure of glucose control in elderly body, and ultimately the onset and development of age-related diabetes.

Type 2 diabetes is a complex systemic syndrome, with impacts on a wide spectrum of organs and tissues. Mounting pieces of evidence discloses that its morbidity increases with age. Although increasing number of studies have been carried out, aiming to disclose possible mechanistic interpretation for age-related T2D, our knowledge about it is still so limited. Taken together the up to date researches, we draw a conclusion that with advancing age, the pathological alterations of pancreatic β cells are supposed to be the key contributor to age-related T2D, and the decreased proliferation and regeneration potential, disturbed transcriptome and proteostasis, increased senescent cell accumulation and the influence of systemic environmental stress may lead to the loss of functional β cell mass and ultimately deficient insulin secretion and insulin action (Figure 2).

## Perspective

Since the pathogenesis of aging is complicated, it is reasonable to think age-related diabetes is a result of gradual accumulation of multiple abnormalities in metabolic system including β cells. A great amount of studies indicated that elderly individuals secrete more insulin to response the glucose challenge, suggesting that β cell compensation occurs in the early age, while the declined number of baseline and compensatory β cells with age, contributes to the onset and progression of T2D. But the process of β cell compensation to compensation failure is still unclear.

It is worth noting that the function and architecture heterogeneity of β cells is reported in both rodents and human, and the reprogramming of β cell heterogeneity also exists in the process aging. Therefore, it is hard to simply explain the occurrence of age-related diabetes just by a general β cell aging but ignoring the complicated heterogeneity in islets.

At present, due to the limitation of human pancreas samples, the animal models used to study the relationship between natural aging and age-related diabetes are mainly focusing on rodents, which still has big gap with human aging in some degree. To further study the pathogenesis of this disease, more ideal animal models and more new technology such as single-cell sequencing and multi-omics studies focusing on β cell function and glucose homeostasis would help to enlarge our limited knowledge about β cell aging and reveal its relationship with the increasing prevalence of age-related diabetes.
